# Risk factors of transplant renal artery stenosis in kidney transplant recipients

**DOI:** 10.1016/j.clinsp.2022.100087

**Published:** 2022-08-02

**Authors:** Gabriel Kanhouche, Gustavo Rocha Feitosa Santos, Henry Campos Orellana, Attilio Galhardo, Ana Carolina Buso Faccinetto, Manoela Linhares Machado Barteczko, Luiz Sérgio F. de Carvalho, Julia Bernardi Taddeo, Renato Demarchi Foresto, Valdir Ambrósio Moises, Helio Tedesco-Silva, José Medina Pestana, Adriano Henrique Pereira Barbosa

**Affiliations:** a Cardiology Division, Hospital São Paulo, Universidade Federal de São Paulo, São Paulo, SP, Brazil; b Nephrology Division, Hospital do Rim, Universidade Federal de São Paulo, São Paulo, SP, Brazil; c Directory of Research and Innovation, Institute for Strategic Management in Healthcare

**Keywords:** Transplant renal artery stenosis, Renal angiography, Risk factors for TRAS, Kidney transplant

## Abstract

**Background:**

Transplant Renal Artery Stenosis (TRAS) is a recognized vascular complication after kidney transplantation. The overall risk predictors of TRAS are poorly understood.

**Methods:**

Retrospective analysis of patients with suspected TRAS (Doppler ultrasound PSV > 200 cm/s) who underwent angiographic study in a single center between 2007 and 2014. All patients with stenosis > 50% were considered with TRAS. Stenosis restricted in the body of the artery was also analyzed in a subgroup.

**Results:**

274 patients were submitted to a renal angiography and 166 confirmed TRAS. TRAS group featured an older population (46.3 ± 11.0 vs. 40.9 ±14.2 years; p = 0.001), more frequent hypertensive nephropathy (30.1% vs. 15.7%; p = 0.01), higher incidence of Delayed Graft Function (DGF) (52.0% vs. 25.6%; p < 0.001) and longer Cold Ischemia Time (CIT) (21.5 ± 10.6 vs. 15.7 ± 12.9h; p < 0.001). In multivariable analyses, DGF (OR = 3.31; 95% CI 1.78‒6.30; p < 0.0001) was independent risk factors for TRAS. DM and CIT showed a tendency towards TRAS. The compound discriminatory capacity of the multivariable model (AUC = 0.775; 95% CI 0.718‒0.831) is significantly higher than systolic blood pressure and creatinine alone (AUC = 0.62; 95% CI 0.558–0.661). In body artery stenosis subgroup, DGF (OR = 1.86; 95% CI 1.04‒3.36; p = 0.03) and Diabetes Mellitus (DM) (OR = 2.44; 95% CI 1.31‒4.60; p = 0.005) were independent risk factors for TRAS.

**Conclusion:**

In our transplant population, DGF increased more than 3-fold the risk of TRAS. In the subgroup analysis, both DGF and DM increases the risk of body artery stenosis. The addition of other factors to hypertension and renal dysfunction may increase diagnostic accuracy.

**TRAS Trial registred:**

clinicaltrials.gov (n° NCT04225338).

## Highlights

• TRAS is the most common vascular complication after kidney transplantation.
• Delayed graft function increases more than 3 times the chance of developing TRAS.
• Diabetes mellitus and DGF are independent risk factors for post-anastomotic TRAS.

## Introduction

Transplant Renal Artery Stenosis (TRAS) is a recognized vascular complication after kidney transplantation defined as the angiographic evidence of transplant renal artery narrowing > 50%.^1^^,^^2^ TRAS accounts for 75% of the vascular events occurring in the post-transplantation period, affects up to 23% of kidney transplant recipients, and is associated with poor long-term patient and allograft survival.^2^^,^^3^^,^^6^ Surgical technique improvement had mitigated perioperative complications, although renal dysfunction and early graft loss have still been documented due to vascular events.^4^, ^5^, ^6^

This vascular involvement is often asymptomatic, but new hypertension, edema, and renal dysfunction are its main clinical manifestations. The clinical suspicion is further corroborated by Doppler Ultrasound indicating decreased blood flow in the transplant renal artery with a peak systolic velocity > 200 cm/s. The gold-standard treatment option to restore kidney perfusion is the percutaneous transluminal angioplasty with the placement of a stent.^7^

The main risk factors associated with TRAS, reported in small cohorts, are elderly recipients, Delayed Graft Function (DGF), Cytomegalovirus (CMV) infection, Diabetes Mellitus (DM), acute rejection, and increased Cold Ischemia Time (CIT).^7^^,^^8^

Although some risk factors are well defined in other studies, there is still a considerable proportion of patients with clinical suspicion who are unnecessarily referred to angiography. The aim of this study is to assess the risk factors for TRAS in suspicious patients for this comorbidity, in a single-center large cohort. By selecting the patient more accurately, it is possible to reduce costs and avoid exposing the patients to unnecessary exams.

## Methods

### Study design

This single-center retrospective study includes data from all adult kidney transplant recipients with suspected TRAS who were referred for angiography at Hospital do Rim between January 2007 and December 2014. The clinical research developed, used the medical records of the Hospital do Rim patients and tabulated data from the Collaborative Transplant Study (CTS).

### Patient selection

Patients with clinical suspicion of TRAS (worsening ambulatory measurement of arterial hypertension despite the use of medications; requiring more antihypertensive drug classes and/or increase > 30% of serum creatinine with other causes of renal dysfunction discarded), were submitted to Doppler Ultrasound of the transplanted kidney artery. Patients with Peak Systolic Velocity (PSV) > 200 cm/s measured by Doppler ultrasound were referred for renal angiographic confirmation. Patients with angiographic stenosis > 50% were considered to have TRAS.^2^

Lesions were differentiated according to their location, in the iliac artery, in the graft anastomosis, renal artery body, renal artery branches, and polar arteries. In order to differentiate possible interferences of surgical techniques, an analysis of a subgroup only with post-anastomotic lesions in the artery body was also performed.

### Statistical analysis

The authors used multiple imputations (*mice* package in R) to handle Missing Values (MV). The authors used a predictive mean matching model for numeric variables, logistic regression (logreg) for binary variables (with 2 levels), and Bayesian polytomous regression (polyreg) for factor variables (≥2 levels). The authors did not impute missing values for the outcomes. The imputation step resulted in 5 complete data sets, each of which contains different estimates of the missing values for all 274 patients in the TraSStudy cohort. After imputation, the authors pooled and merged all 5 datasets to perform stepwise logistic regressions. Sensitivity analyses were conducted in each of the generated datasets.

Normally distributed data were presented as mean ± SD and skewed data as median (Interquartile Range [IQR]). Normality of distribution and variances were checked using histograms, the Kolmogorov-Smirnoff test, normal probability plots and residual scatter plots. Chi-Square or Kruskal-Wallis or two-tailed *t*-tests were used for comparison of baseline data. Logistic regression models were done to identify risk factors associated with > 50% TRAS, using the Odds Ratio (OR) and 95% Confidence Intervals (95% CI) to estimate the relative risk. Regression models were built by using a stepwise approach, limiting to 11 variables per step or per model since the authors found 108 individuals with non-TRAS and 166 with TRAS. A sub-group analysis was performed in patients with lesions restituted to the artery body, excluding those with ostial, distal branches, and iliac artery lesions.^9^^,^^10^ The discriminatory ability of the models was assessed using the Area Under the Receiver Operating Characteristic Curve (AUROC); p-values < 0.05 were considered statistically significant. Analyses were carried out using R(v3.5.3).

## Results

### Baseline patient characteristics

**Fig. 1:**
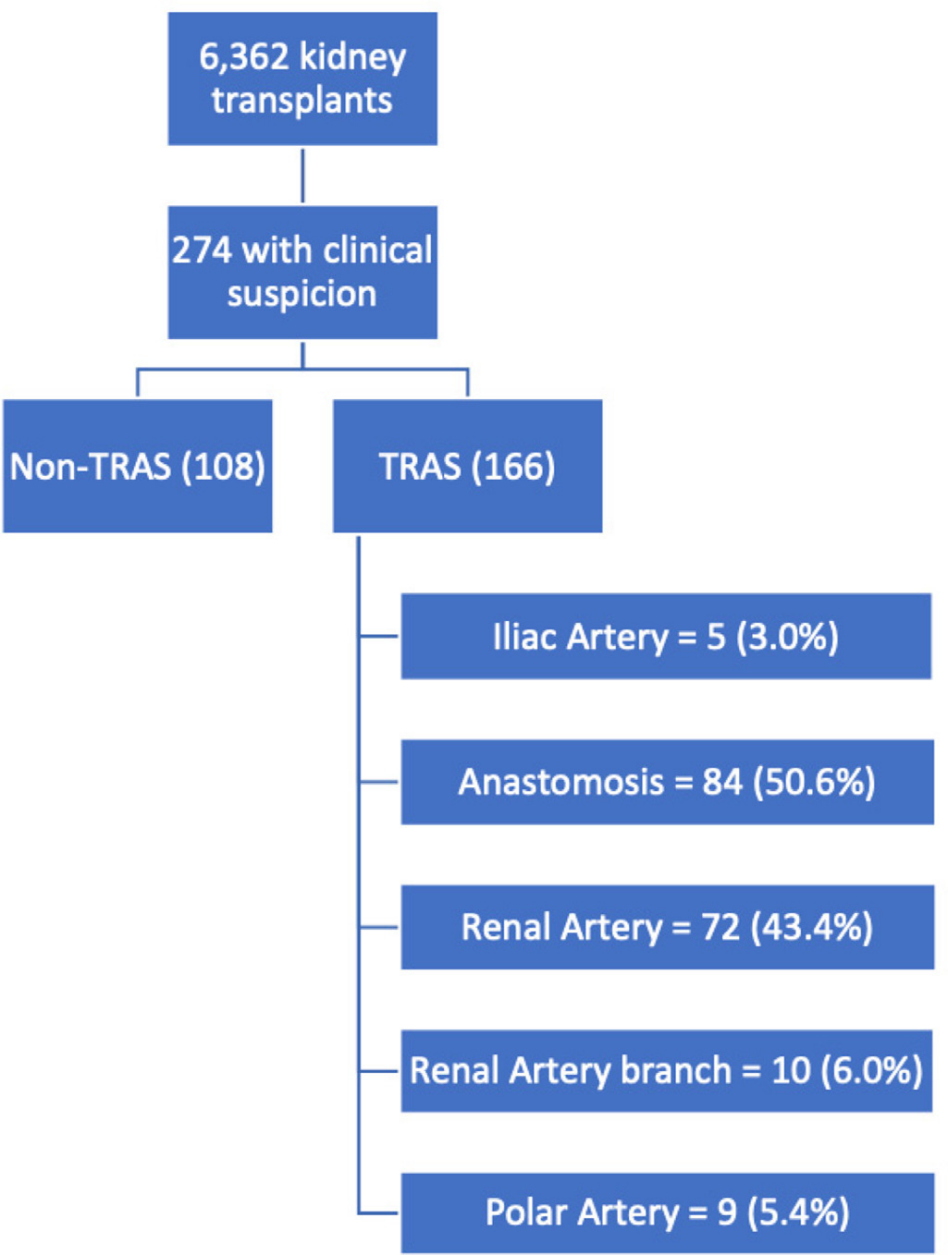
Flowchart.

During this period, 6,362 kidney transplants were performed at Hospital do Rim, 274 (4.3%) of them had clinical suspicion of TRAS. After the arteriography, 166 (60.6%) cases were confirmed with a diagnosis of TRAS (Fig. 1). Both groups have very similar clinical and demographic characteristics of recipients. The proportion of gender, ethnicity and comorbidities such as hypertension, dyslipidemia, and smoking did not differ between groups. Despite this, the most frequent etiology of CKD in TRAS group was hypertension nephropathy (30.1% vs. 15.7%, p = 0.01). This group also had older recipients (46.3 ± 12.0 vs. 40.9 ± 14.2 years old, p < 0.001), more diabetic (31.5% vs. 20.6%, p = 0.06), and shorter stature recipients (167 ± 8.6 vs. 170 ± 9.7 cm, p = 0.02) than the control group (Table 1).

**Table 1:** Demographic characteristics.

	Non-TRAS (108)	TRAS (166)	p
Recipient gender, female, n (%)	20 (18.5)	35 (21.1)	0.71
Recipient age, years, mean ± SD	40.9 ± 14.2	46.3 ± 12.0	0.001
Recipient ethnicity, n (%)			0.23
Caucasian	44 (41.5)	61 (37.0)	
Black	14 (13.2)	32 (19.4)	
Asian	1 (0.9)	4 (2.4)	
Native indian	30 (28.3)	53 (32.1)	
Others	17 (16.0)	15 (9.1)	
BMI, kg/m^2^, mean ± SD	24.2 ± 4.6	24.9 ± 4.2	0.31
Height (cm, D0) (mean (SD))	170.43 (9.73)	167.27 (8.63)	0.02
Hypertension, n (%)	91 (85.0)	151 (91.0)	0.19
Diabetes mellitus, n (%)	22 (20.6)	52 (31.5)	0.06
Dyslipidemia, n (%)	14 (13.0)	19 (11.6)	0.88
Smoking, n (%)	10 (9.4)	16 (10.0)	1.00
CKD etiology, n (%)			
Hypertensive nephropathy	17 (15.7)	50 (30.1)	0.01
Diabetic nephropathy	17 (15.7)	37 (22.3)	0.24
Polycystic nephropathy	7 (6.5)	7 (4.2)	0.58
Glomerulonephritis	21 (19.4)	24 (14.5)	0.35
Other diagnoses	9 (8.3)	9 (5.4)	0.48
Unknown	43 (39.8)	54 (32.5)	0.27
CMV + serology, n (%)	89 (87.3)	145 (90.1)	0.61
Time on dialysis, months, mean ± SD	39.1 ±30.7	41.3 ±34.2	0.59
Type of treatment, hemodialysis, n (%)	100 (98.0)	157 (95.2)	0.30
Donor Age, years, mean ± SD	44.3 ± 13.9	46.3 ± 13.9	0.26
Donor type, n (%)			<0.001
Living	45 (41.7)	31 (18.8)	0.007
Deceased (Standard criteria)	40 (37.0)	95 (57.6)	0.012
Deceased (Expanded criteria)	23 (21.3)	39 (23.6)	
Brain death etiology, n (%)			0.46
Trauma	19 (31.7)	35 (25.7)	
Neurological	27 (45.0)	77 (56.6)	
Cardiovascular	13 (21.7)	21 (15.4)	
Others	1 (1.7)	3 (2.2)	
Mismatch 4‒6, n (%)	19 (18.6)	28 (17.7)	0.98
CIT, hours, mean ± SD	15.7 ± 12.9	21.5 ± 10.6	<0.001
Treatment for acute rejection, n (%)	36 (34.3)	60 (37.7)	0.66

TRAS, Transplant Renal Artery Stenosis; SD, Standard Deviation; BMI, Body Mass Index; CKD, Chronic Kidney Disease; CMV, Cytomegalovirus; CIT, Cold Ischemia Time.

Most of the recipients were submitted to hemodialysis before transplantation and time on dialysis was similar between the groups (41.3 ± 34.2 vs. 39.1 ± 30.7 months, p = 0.59). Living donor transplant was more prevalent in the non-TRAS group (41.7% vs. 18.8%, p = 0.007) and consequently, CIT was higher in the TRAS group (21.5±10.6 vs. 15.7±12.9 hours; p < 0.001) (Table 1).

### Pre-arteriography data

The median time between the transplant and arteriography was 5 months in the TRAS group and 6 months in the control group (Table 2). Prior to performing the arteriography, despite both groups having a similar number of anti-hypertensive drugs (2.2±1 vs. 2.2±1 drugs, p = 0.88), patients in the TRAS group had mean ambulatory SBP (154.6±24.7 vs. 144.1±23.4 mmHg, p < 0.001), DBP (92.8 ± 16.3 vs. 88.3 ± 17.4 mmHg, p = 0.03) and serum creatinine (2.1 [1.7, 3.2] vs. 1.9 [1.6, 2.5] mg/dL, p = 0.01) higher than those in the control group. In accordance, the eGFR is lower in the TRAS group. As expected, the patients that need intervention (TRAS group) had higher PSV (428.6±151.0 vs. 343.2±113.5 cm/s, p < 0.001).

**Table 2:** Pre-arteriography data.

	Non-TRAS (108)	TRAS (166)	p
Time post transplantation, months, median (IQR)	6.0 (3.0, 17.0)	5.0 (3.0, 10.0)	0.27
Serum creatinine, mg/dL, median (IQR)	1.9 (1.6, 2.5)	2.1 (1.7, 3.2)	0.01
eGFR, median (IQR)	42 (14, 61.5)	34 (9, 50)	0.003
SBP, mmHg, mean ± SD	144.1 ± 23.4	154.6 ± 24.7	<0.001
DBP, mmHg, mean ± SD	88.3 ± 17.4	92.8 ± 16.3	*0.03*
Total cholesterol, mg/dL, mean ± SD	183.7 ± 49.1	179.8 ± 46.7	0.54
Anti-hypertensive drugs, mean ± SD	2.18 ± 0.99	2.16 ± 1.07	0.88
HDL-C, mg/dL, mean ± SD	42.5 ± 12.1	43.6 ± 11.6	0.47
LDL-C, mg/dL, mean ± SD	110.8 ± 42.1	105.4 ± 37.2	0.32
CMV infection, n (%)	26 (24.5)	55 (33.3)	0.159
PSV cm/s, mean ± SD	343.2 ± 113.5	428.6 ± 151.0	<0.001
DGF, n (%)	22 (25.6)	65 (52.0)	<0.001
Retransplant, n (%)	3 (2.8)	10 (6.1)	0.33
Immunosuppressive regime, n (%)			0.46
TAC, MPS, Pred	30 (29.4)	61 (39.9)	
TAC, AZA, Pred	43 (42.2)	55 (35.9)	
CsA, AZA, Pred	21 (20.6)	29 (19.0)	
CsA, MPS, Pred	4 (3.9)	4 (2.6)	
Others	4 (3.9)	4 (2.6)	

IQR, Interquartile Interval; SD, Standard Deviation; SBP, Systolic Blood Pressure; DBP, Diastolic Blood Pressure; HDL-C, High Density Lipoprotein – Cholesterol; LDL-C, Low Density Lipoprotein – Cholesterol; CMV, Cytomegalovirus; PSV, Peak Systolic Velocity; DGF, Delayed Graft Function; TAC, Tacrolimus; MPS, Mycophenolate Sodium; AZA, Azathioprine; Pred, Prednisone.

Although the TRAS group had a higher prevalence of patients infected with CMV, there was no statistically significant difference between the groups (Table 2). The cholesterol collected in the immediately previous consultation of the arteriography was also analyzed, and no difference was observed.

In the TRAS group, the prevalence of patients that developed DGF after transplant surgery was higher (52.0% vs. 25.6%, p < 0.001). Despite the higher prevalence of patients undergoing retransplant in the TRAS group (6.1% vs. 2.8%, p = 0.33), this variable was not statistically significant in the univariate analysis.

Most patients used an immunosuppression regimen that included Tacrolimus, Prednisone, and Azathioprine or Mycophenolate. The TRAS group had a higher percentage of triple treatment that included Mycophenolate, but there was no statistical significance.

### Multivariable analysis

In order to identify the independent factors for TRAS in suspicious patients, a stepwise method was performed. As a data-driven method, data on height, diabetes, serum creatinine, HDL-cholesterol, time since transplantation, peritoneal dialysis, DGF, CIT, SBP, and hypertensive nephropathy prior to transplantation were incorporated into the multivariate analysis model (Table 3). As expected, creatinine and SBP were shown to be relevant to predicting TRAS. It was also possible to analyze that peritoneal dialysis and DGF are risk factors, the latter with an increased chance of more than 3 times for TRAS. Meanwhile, height proved to be a protective factor. Although not significant, DM and CIT showed a tendency towards TRAS. Of note, the compound discriminatory capacity of this 10-variable model (AUC = 0.775; 95% CI 0.718‒0.831) is significantly higher than systolic blood pressure and creatinine alone (AUC = 0.62; 95% CI 0.558–0.661).

**Table 3:** Multivariable analysis of risk factors for TRAS.

	OR	95% CI		p
		Lower	Upper	
**Height (per 1 cm)**	0.97	0.94	0.99	0.048
**Diabetes mellitus**	1.80	0.95	3.48	0.074
**Creatinine (per 1 mg/dL)**	1.15	1.02	1.32	0.035
**HDL (per 1 mg/dL)**	1.01	0.99	1.04	0.233
**Time since Transplantation (per 1 month)**	0.99	0.98	1.00	0.210
**Peritoneal Dialysis**	12.32	1.77	252.79	0.029
**Delayed graft function**	3.31	1.78	6.30	0.0001
**Cold ischemia time (h) (per 1h)**	1.02	0.99	1.04	0.092
**Systolic blood pressure at arteriography (per 1 mmHg)**	1.02	1.01	1.03	0.002
**Hypertensive nephropathy**	1.70	0.86	3.45	0.134

The best model fit obtained an Area Under the Curve (AUC) in Receiver Operator Characteristic (ROC) curve of 0.7745 (95% CI 0.718‒0.831).

As seen in Fig. 1, most lesions were present in the anastomosis or in the body of the lesion. Then, a sub-analysis of post-anastomotic lesions in the renal artery body was performed. The multivariate model included height, diabetes mellitus, dyslipidemia, time since transplantation, DGF, CIT, SBP, and glomerulonephritis etiology prior to transplantation (Table 4). It is possible to observe that both diabetes mellitus and DGF were shown to be independent risk factors for -post-anastomotic TRAS.

**Table 4:** Multivariable analysis of risk factors for post anastomotic TRAS.

	OR	95% CI		p
		Lower	Upper	
**Height (per 1 cm)**	0.97	0.94	1.00	0.07
**Diabetes mellitus**	2.44	1.31	4.60	0.005
**Dyslipidemia**	0.45	0.16	1.12	0.100
**Time since Transplantation (per 1 month)**	0.98	0.97	1.00	0.086
**Delayed graft function**	1.86	1.04	3.36	0.038
**Cold ischemia time (h) (per 1h)**	1.02	0.99	1.05	0.190
**Systolic blood pressure at arteriography (per 1 mmHg)**	1.01	0.99	1.02	0.142
**Glomerulonephritis prior to Transplantation**	1.49	0.70	3.12	0.287

The best model fit obtained an Area Under the Curve (AUC) in Receiver Operator Characteristic (ROC) curve of 0.7149 (95% CI 0.6508‒0.7789).

## Discussion

The present study's population sample has similar characteristics compared to previously published cohorts. In our center, 2.6% of kidney transplanted recipients had a diagnosis of TRAS, which is in line with that found in the literature of 1%‒23%.^3^ The mean time for diagnosis of TRAS was 5 months, in concordance with the literature (3 months to 2 years).^11^ Chen et al.^12^ published in 2015 the pathophysiology and temporality of the injuries, associating the earliest injuries to complications of the surgical technique and the graft. TRAS represents an important vascular complication in patients with renal graft with risk factors and clinical signs similar to native kidney stenosis such as hypertension, increased number of antihypertensive drugs, high PSV at Doppler, and worsening renal function. However, some small articles suggest an immunological role that is not yet consensual.^13^ Despite clinical and ultrasound suspicion, 39% of the patients referred for arteriography did not meet the criteria for TRAS.

In both groups, it is possible to observe an average of PSV much higher than the cut adopted. In a systematic review, there are authors who adopt the PSV cut between 200‒300 cm/s.^1^ Even if the criterion were stricter, it would increase specificity and decrease sensitivity, keeping the PSV mean at non-TRAS (343.2±113.5 cm/s) higher than the cutoff. The authors know that doppler is an examiner-dependent method, and even in a center as specialized as ours, measurement failures can occur. Fananapazir et al.^7^ proposed auxiliary ultrasound analyses to increase specificity without decreasing sensitivity. Even in mild stenosis, the average PSV was greater than 400 cm/s in this study.

Diabetes is a known risk factor for atherosclerosis, due to the greater endothelial permeability to lipid macromolecules in the coronary arteries, which can compromise other vascular beds, including renal arteries. Willicombe et al.^14^ described that diabetes represents an odds ratio of 3.2 for TRAS with a post-stenotic lesion. A previous study by Hurst et al.,^11^ with a larger population sample, did not show statistical significance in the multivariate analysis, despite the difference in prevalence between the groups. In the present study, the proportion of diabetes between the groups (31.5% vs. 20.6%; p = 0.06) is very similar to the study by Willicombe, which evaluates post-anastomotic injuries. When the authors selected only patients with stenosis in the artery body, excluding ostial, iliac and distal lesions, it is possible to say that diabetes has a high risk of TRAS, in agreement with the literature (Table 4). The authors believe to be explained by the endothelial injury caused by diabetes in the atherosclerotic mechanism in the body of the artery as it occurs in other vessels, mainly coronary.^14^

As expected, pre-arteriography serum creatinine, systolic blood pressure, and diastolic blood pressure were significantly different between both groups.^15^^,^^16^ These manifestations, triggered by the renin-angiotensin-aldosterone system, are the first clinical signs that can raise suspicion of TRAS. Systolic blood pressure was shown to be an independent risk factor for TRAS with statistical significance (Table 3). The high blood pressure levels create a shear load on the luminal wall with endothelial damage, the appearance of inflammatory and prothrombotic factors leading to luminal reduction.^11^ Therefore, it is difficult to differentiate the cause or consequence effect from systolic pressure. It presents in the initial clinical manifestations of the pathology and the high pressure perpetuates vascular endothelial damage.

Audard et al.^8^ compared the presence and absence of delayed graft function with a 4.61 times greater risk of developing TRAS. In the present study, this variable was also confirmed as a risk factor with an increased risk of developing TRAS (OR = 3.30; 95% CI 1.78‒6.30; p < 0.0001) in this patient profile. According to the study by Halimi et al.,^17^ the increased period of ischemia can cause vascular, endothelial, and parenchymal damage leading to delayed graft function due to the production of oxygen-free radicals. Reactive oxygen species can influence vascular tonicity and induce inflammatory processes. It is observed that in the TRAS group the patient who received an organ from a deceased donor is more common (81.9% vs. 56.5%; p < 0.001), and as a consequence was submitted to a longer period of cold ischemia time. In agreement with the other studies, CIT also presented a tendency to risk factors (OR = 1.02; 95% CI 0.99‒1.04; p = 0.09).^8^^,^^18^^,^^19^

Despite some studies pointing to CMV infection as an independent risk factor for TRAS, the present study did not confirm the same result. Audard et al.^6^ reported that the average time between CMV infection and diagnosis of TRAS was 380 days. This also diverged from our population, which had an average time of 206 days. Evidence from previous literature consolidates this variable as an important predictor of TRAS due to its immunological role. The proportion of infection in both groups is very similar to previous studies that identified this variable as a risk factor. It is possible to observe a higher prevalence of patients infected with CMV in the TRAS group, but there was no statistical significance in the present study. There was also no correlation with seropositivity (Immunoglobulin G) for CMV, found in 90% of pre-transplant patients who developed TRAS.^20^

In multivariate analysis, peritoneal dialysis was also demonstrated as an independent factor for TRAS, however, the authors believe that due to the low representativeness of this condition in relation to hemodialysis, it may have generated a sampling bias. Therefore, the authors have no scientific basis to justify this variable as statistically representative. The same occurs with height, which was shown to be a protective factor. In view of the current literature and the available knowledge, the authors have not found justification to explain this finding. The stepwise model mathematically selects data variables and disregards previous knowledge, thereat those variables were included in the model based on their initial statistical weight.

The accuracy of the variables found in Table 4 to predict TRAS (AUC = 0.77; 95% CI 0.718‒0.831), is much higher than that of classic factors such as hypertension and renal function alone (AUC = 0.62; 95% CI 0.558–0.661). Thus, a future opportunity arises to create a Score to identify TRAS more accurately. It is evident that factors such as DGF, DM, and CIT should be added to hypertension and renal function in clinical practice to investigate this potentially serious complication in a more accurate way to avoid unnecessary exams.

This study has limitations, despite presenting data from a large volume center. The main limitation is that it is a retrospective and single-center study. However, it is unlikely that prospective randomized studies in the scenario of TRAS involving intervention are feasible, given the complications resulting from graft stenosis and consequent renal loss. Some donor information was not available for being collected in the present study's registry.

## Conclusion

Thus, the authors can conclude that the well-established criteria for TRAS as risk factors such as creatinine and arterial hypertension were present in this study. DGF and diabetes also showed a strong correlation for the appearance of TRAS, as already described in smaller studies. Although the authors have one of the largest series on the subject, it is clear that there is a need for even greater multi-centers studies to clarify some controversial points such as CMV infection, acute rejection, and CIT. These variables tended to be a risk factor but were not statistically significant in the present study.

## Disclosure

Helio Tedesco Silva Junior has received research grants and travel and consulting honoraria from Novartis, Sanofi and Pfizer. The remaining authors have no conflicts of interest to disclose.

## Authors’ contributions

Gabriel Kanhouche: Study design, data acquisition, statistical analysis and writing of the manuscript.

Gustavo Rocha Feitosa Santos, Henry Campos Orellana, Attilio Galhardo, Ana Carolina Buso Faccinetto, Manoela Linhares Machado Barteczko, Julia Bernardi Taddeo: Data acquisition.

Luiz Sérgio F. de Carvalho: Statistical analysis and writing of the manuscript.

Renato Demarchi Foresto: Writing and revision of the manuscript.

Valdir Ambrósio Moises, Helio Tedesco-Silva, José Medina Pestana: Study design, writing and revision of the manuscript.

Adriano Henrique Pereira Barbosa: Study design, data acquisition, statistical analysis, writing and revision of the manuscript.

## Declaration of Competing Interest

The authors declare no conflicts of interest.
